# Effects of engaging communities in decision-making and action through traditional and religious leaders on vaccination coverage in Cross River State, Nigeria: A cluster-randomised control trial

**DOI:** 10.1371/journal.pone.0248236

**Published:** 2021-04-16

**Authors:** Angela Oyo-Ita, Xavier Bosch-Capblanch, Amanda Ross, Afiong Oku, Ekpereonne Esu, Soter Ameh, Olabisi Oduwole, Dachi Arikpo, Martin Meremikwu

**Affiliations:** 1 Department of Community Medicine, Faculty of Medicine, College of Medical Sciences, University of Calabar, Calabar, Cross River State, Nigeria; 2 Effective Health Care Alliance Programme, Institute of Tropical Disease, Research and Prevention, University of Calabar Teaching Hospital, Calabar, Cross River State, Nigeria; 3 University of Basel, Basel, Switzerland; 4 Swiss Tropical and Public Health Institute, Basel, Switzerland; 5 Department of Public Health, Faculty of Allied Medicine, College of Medical Sciences, University of Calabar, Calabar, Cross River State, Nigeria; 6 Department of Global Health and Population, Harvard T.H. Chan School of Public Health, Harvard University, Boston, Massachusetts, United States of America; 7 Department of Medical Laboratory Science, Achievers University, Owo, Ondo State, Nigeria; 8 Department of Paediatrics, Faculty of Medicine, College of Medical Sciences, University of Calabar, Calabar, Cross River State, Nigeria; IAVI, UNITED STATES

## Abstract

**Background:**

Vaccination coverage levels fall short of the Global Vaccine and Action Plan 90% target in low- and middle- income countries (LMICs). Having identified traditional and religious leaders (TRLs) as potential public health change agents, this study aimed at assessing the effect of training them to support routine immunisation for the purpose of improving uptake of childhood vaccines in Cross River State, Nigeria.

**Methods:**

A cluster-randomised controlled study was conducted between 2016 and 2019. Of the 18 Local Government Areas (LGA) in Cross River State, eight (four urban and four rural LGAs) were randomized into the intervention and control study arms. A multi-component intervention involving the training of traditional and religious leaders was implemented in the four intervention LGAs. Baseline, midline and endline surveys collected information on children aged 0–23 months. The effect of the intervention on outcomes including the proportion fully up-to-date with vaccination, timely vaccination for pentavalent and measles vaccines, and pentavalent 1–3 dropout rates were estimated using logistic regression models using random effects to account for the clustered data.

**Results:**

A total of 2598 children at baseline, 2570 at midline, and 2550 at endline were included. The intervention was effective in increasing the proportion with at least one vaccine (OR 12.13 95% CI 6.03–24.41p<0.001). However, there was no evidence of an impact on the proportion of children up-to-date with vaccination (p = 0.69). It was effective in improving timeliness of Pentavalent 3 (OR 1.55; 95% CI: 1.14, 2.12; p = 0.005) and Measles (OR 2.81; 96% CI: 1.93–4.1; p<0.001) vaccination. The odds of completing Pentavalent vaccination increased (OR = 1.66 95% CI: 1.08,2.55).

**Conclusion:**

Informal training to enhance the traditional and religious leaders’ knowledge of vaccination and their leadership role can empower them to be good influencers for childhood vaccination. They constitute untapped resources in the community to boost routine immunisation.

Pan African Clinical Trial Registry (PACTR) PACTR202008784222254.

## Introduction

### Background

In an effort to reduce childhood morbidity and mortality, the World Health Organization (WHO) launched the Expanded Program on Immunisation (EPI) in 1974, targeting the six childhood killer diseases (polio, tetanus, diphtheria, pertussis, measles, and tuberculosis). These diseases together contributed more than one-third of childhood deaths globally in 2008 [1]. Vaccines prevent 2–3 million childhood deaths globally [2] via individual protection and, if coverage is sufficient, herd immunity.

Overall, there has been a global increase in vaccination rates in the past three decades [3]. Coverage levels however still fall short of the Global Vaccine and Action Plan (GVAP) target of 90% and are much lower in low- and middle- income countries (LMICs). Nigeria is one of the ten countries with coverages of third dose of Diphtheria, Tetanus, Pertussis (DTP3) and the first dose of measles both below 50% [4]. The 2016/2017 Multi-Indicators Cluster Survey (MICS) in Nigeria estimated that the full vaccination coverage of children aged 12–23 months was 21% [5].

The reasons for low vaccination coverage are related to immunisation systems, family characteristics, parental knowledge and attitude, and limitations with the communication of immunisation information [3]. Stemming from the multi-factorial reasons behind non- and under-vaccination of children, Rainey et al suggested a multi-faceted approach to bridge the gaps [3].

Interventions focusing on traditional leaders as agents of change have been used to address public health challenges. A report on engagement of traditional leaders in community health from John Hopkins University stated that traditional leaders in Zambia were “an untapped resource and a key link needed to bring various stakeholders on the same path to better health” [6]. Traditional leaders have had a positive influence on HIV and AIDS prevention in South Africa [7] and supported the scaleup of polio campaigns in northern Nigeria [8]. Traditional and Religious Leaders (TRLs) are known to be influential and are respected in their communities as opinion formers and guides in religious, social and family life. They have been used to increase use of community health services. Community members hold them in high esteem and depend on them, to a large extent, to make decisions [9].

In Nigeria, traditional leaders lead the ward development committees, which enable community participation within the primary healthcare system. The role of the ward development committee includes identifying health needs and mobilizing resources to meet these needs. We developed, implemented, and evaluated a multi-faceted intervention which used Traditional and Religious Leaders (TRLs) to increase the uptake of Routine Immunisation (RI) within the primary healthcare system.

### Objectives

The objectives of the trial were to evaluate the impact of the intervention on vaccination coverage in children 0–23 months old, the drop-out rate for Pentavalent 3 vaccine, the timing of Pentavalent and measles vaccination, the morbidity and mortality of vaccine-preventable diseases, and the utilization of other preventive clinic services. Other outcomes studied were changes in the processes and perceptions of actors and the cost-effectiveness of the intervention. This report is focused on the quantitative components of the study.

## Methods

### Trial design

This was a cluster-randomised controlled trial. The unit of randomisation was the Local Government Area (LGA), since TRL activities were coordinated at this level. Eight LGAs were randomized to the intervention or control arms. The baseline survey was carried out in December 2016. The training for the intervention took place in May 2017 in the intervention LGAs only. The mid-line survey took place in February 2018 and the final survey in January 2019.

### Study setting

The study setting was Cross River State in the south-south geopolitical zone of Nigeria. It is one of the 36 States in Nigeria with approximately three million people. The State is divided into three senatorial districts: the northern, central and southern. There are 18 Local Government Areas (LGAs) which are unevenly distributed in the three senatorial districts of the State: south (seven LGAs), central (six LGAs) and north (five LGAs). Each LGA is further subdivided into Wards, with10 to 13 Wards in each LGA. Each Ward is comprised of villages. Every village is led by a village head (a traditional leader). Every Ward has a clan head, selected among the village heads within the Ward. The village heads, with their respective clan head, constitute the Council of Chiefs in each Ward.

Local Government Areas were eligible to be included in the study if they had a Primary Health Care Centre, and Ward Development Committees, and if they had no security issues. Eight were included in total, with four intervention and four control.

The study team paid courtesy visit to the selected participants for the training in the company of the Ward Focal Person. They were presented with an invitation letter and the purpose of the intervention explained to them.

### Intervention

The intervention was carried out between May 2017 and November 2018). It had multiple components which were designed to fit the structure of the primary healthcare system via the ward development committee. The Committee was composed of members of the community including traditional and religious leaders (TRL). The ward development committees provide oversight to the primary healthcare facilities.

The intervention included training of the TRLs as a means of improving their leadership role in the community and in the ward development committee and their knowledge and perspective on vaccination. The components of the intervention were: TRL training, Health workers’ training, community engagement and strengthening of the ward development committee.

The preparatory phase lasted for five months during which the training tools were prepared, and pilot tested. The training tools were developed by the research team by adapting existing national and international tools [10, 11]. The adapted tools were reviewed by the training team who comprised four retired Community Health Officers who were experienced health educators and community mobilizers, and had worked either as Primary Health Care Coordinators or Social Mobilization Officers. Pilot testing of the tools was carried out in one of the Local Government Areas in the State that was not included in the study, with five traditional and three religious leaders in attendance. The aims were to test the ability of the trainers to communicate the training objectives and to get feedback from the trainees on their understanding of the objectives of the training and the usefulness of the training materials to improve knowledge and perspective on vaccination.

#### Traditional and religious leaders’ training

The training was conducted at the LGA level for TRLs from the selected villages. A total of 23 participants were trained in each of the four intervention LGAs. This comprised all the village heads in the selected villages and the Clan Head from the selected Wards and two religious leaders from each Ward. None of the selected participants held dual offices. The majority of the religious groups were Christians; only Obudu, an LGA in the northern senatorial district, had an Islamic religious leader. Two religious leaders with the largest followers and the leader of the only Islamic group were invited to participate in the training in each Ward.

The venue of the training was the primary healthcare facility in three LGAs and the town council hall in one LGA. Training sessions included types of leadership, characteristics of a good leader, transformational leadership, effective communication, vaccination, community mobilization, etc. All the participants were literate and could be communicated with in Nigerian Pidgin (also called Nigerian Creole), which is an informal English-based creole language spoken as a *lingua franca* across Nigeria. The sessions were interactive and participatory. Methods of training adopted included brainstorming, large and small group discussions, role-plays, problem-solving case studies, and learning aids. Five sessions of training were held in the first nine months and three sessions in the second nine months. No training was conducted for traditional and religious leaders in the control sites.

#### Health workers’ training

The training was conducted for the health workers in the intervention sites to improve the quality of their summarization and communication of vaccination data with laypersons. The cadres of health workers were the Senior Community Health Extension Workers and Community Health Extension Workers. A one-day training session on data summarization and presentation using infographic aid was held in one of the Health Centres in each intervention LGA. The participants were the health workers in charge of the Health Centres from the three Wards included in the study, the Ward Focal Person, the Local Immunisation Officer, the Monitoring and Evaluation Officer, and the Cold Chain Officer. The training lasted for three hours. Data from the immunisation registers generated from routine services in health facilities were analysed and presented on a dashboard. The dashboard was a portable 60 by 70 cm plastic panel with stick-on plaques for ease of conveyance to meetings outside the health facility. The health workers used this to share data with the TRLs at Council of Chiefs’ meetings and the ward development committee meetings. Data displayed on the dashboard included monthly RI uptake and dropouts on the Pentavalent 3 vaccine. Hands-on training was also conducted for the health workers on keeping a defaulters’ register following a report from them that they did not have a means of identifying children that had dropped out of immunisation. This training was delivered on the fifth month of the intervention. They were also trained on the management of adverse effects of vaccination. No training was conducted for health workers in the control sites.

#### Community engagement

The TRLs in the intervention sites educated their communities during their routine community meetings on vaccination. In addition, vaccination data summarized by the health workers from RI services were displayed on the dashboard and shared during the monthly ward development committee meetings. The religious leaders utilized the church and the mosque to share information with the community. Similar community meetings were held routinely in a monthly basis in the control sites. However, the information on vaccination was not shared.

#### Leadership and coordination of the ward development committee

As at the time of commencement of the intervention, the ward development committees had become inactive in most of the Wards in the intervention sites following non-support of the Committees’ meetings by the government: only 3 of the 12 were functioning. Following the training, the nine non-active ward development committees were reactivated and began holding regular meetings. The ward development committees in the control sites were holding regular meetings devoid of the trial related intervention on vaccination.

### Evaluation of the intervention

#### Outcomes

The primary outcome was the proportion of children aged 0-23months with up-to-date vaccination for BCG, OPV, Pentavalent 3, PCV, measles and yellow fever appropriate for age.

The secondary outcomes were the proportion of children with timely Pentavalent and measles vaccination, the proportion of children who had Pentavalent 1 but not Pentavalent 3 of those who were old enough (target age plus two weeks), trend in the number of children with measles and deaths from the target disease, and trend in ANC, delivery and out-patient attendance from three years prior to the intervention. We defined timely vaccination as receiving the scheduled vaccination no more than two weeks late following Clark and Sanderson, 2009 [12].

#### Randomisation

Randomisation was done at the LGA level. Local Government Areas were stratified by geographical zone and within each stratum allocated to intervention and control arms by simple random sampling using R by the collaborating institution, Swiss Tropical and Public Health institute (Swiss TPH). Eight LGAs were selected with four as intervention and four as control LGAs. The LGAs were stratified by senatorial district and whether they were urban or rural into four pairs. There were two LGAs from the north, three from central and three from the south senatorial districts and four were urban and four rural LGAs and these formed pairs for central rural, north urban, south rural and mixed urban. One LGA from each pair was randomised to the intervention and the other to control arms of the study. Similarly, three Wards were randomly selected from each LGA, and four villages from each Ward making a total of 48 villages per study arm. The selected Wards were non-adjacent to reduce possible contamination.

#### Sample size determination

The sample size calculation was based on the proportion of fully vaccinated children aged 0 to 23 months. We assumed that the pre-intervention proportion of fully vaccinated children was 53% [5] and we wished to detect a change of 10% (i.e. 63% fully vaccinated children post-intervention) with at least 80% power and a 5% significance level. We set the variation between LGA to a value of k (the standard deviation divided by the mean) of 0.18 based on available data on the mean coverage of Pentavalent 3 from routine vaccine records in the study setting. The same value of k was assumed for the variation between Wards and villages. We also allowed for a non-response of 15%. The sample size was determined using a simulation written in R which allowed for the clustering in the sample and the regression analysis. Specifying four Wards per LGA, three villages per Ward and 25 children per village, the smallest number of LGA which gave at least 80% power was four per arm. This gave a total of 1200 children per study arm per survey.

#### Blinding

Blinding of the participants to the intervention was not possible. However, the respondents at the survey and the data collectors were blinded. The respondents were caregivers of children aged 0 to 23 months who were unaware of the training the TRLs received. The data collection was carried out by the Demographic Health Surveillance System team in the University of Calabar, an independent team from the research team.

#### Selection of children for the surveys

Independent household surveys were carried out at baseline, mid-line (after 9 months of intervention) and end-line (after 18 months of intervention).

In each selected village, 25 households with children aged 0–23 months were selected using the WHO spin-the-pen method [13] because there was no list of all households. A team member dedicated to sampling of households went to the centre of the village and spun a bottle to choose a random direction. The “sampler” then walked in the direction indicated until the edge of the village was reached, sketching a map of all the households passed, and numbering them as they went. One of these houses was selected at random as the starting point, or “house 1” of the village. At this house, a bottle was spun to choose a random direction, and the sampler walked in that direction until they came to another household, which was the second house of the village, and so on. If there was a junction in the path, the bottle was spun again to select from the choices available. This procedure was repeated until 25 households with children were counted [14].

#### Questionnaire

A semi-structured interviewer-administered questionnaire was used for the survey on immunisation coverage. The questionnaire was adapted from the WHO vaccination coverage tool [14]. Verbal consent was obtained from the child’s caregiver before applying the questionnaire. Information was collected on household characteristics, immunization status of the child, caregiver’s knowledge of vaccination, prevalence of selected childhood diseases, mother’s health facility utilization, and delivery. Data on the child’s immunization history was extracted from the vaccination card. When this was not available parental recall was used. Open Data Kit (ODK) was used for data collection. Coding of the paper tool into the mobile device included the creation of built-in data validation logic, constraints and loops.

#### District Health Information System (DHIS)

Data from the DHIS was extracted from the selected Wards on facility attendance, attendance at antenatal care, deliveries, and measles from 2014 (three years before the intervention) to 2018 (endline of the intervention) to monitor trend in uptake of services and incidence of vaccine preventable diseases.

### Statistical analysis

The effect of the intervention was estimated using logistic regression, including random effects for village, Ward and LGA was used to account for the non-independence of the clustered observations. We included the baseline survey in the model to allow each village to account for baseline differences. The effect of the intervention was estimated as the additional increase in vaccine uptake between the baseline and either the midline or endline surveys in the intervention arm compared to the control arm. Covariates such as the age of caregiver, residing in a hard-to-reach community, distance to the nearest health facility, hard-to-reach and rural/urban setting were assessed for imbalance at baseline. The analysis was carried out in R.

### Ethical permission and trial registration

Ethical approval was obtained from the Cross River State Ministry of Health (ref: CRS/MH/HREC/016/Vol.V/023) in May 2016.

The abstract of the protocol of this study was published on the website of the Funder (https://www.3ieimpact.org/evidence-hub/impact-evaluation-repository/effects-engaging-communities-decision-making-and-action), therefore, the protocol was not registered before recruitment of the participants into the study. No changes were made to the protocol. There are no ongoing or related trials of this intervention. The trial is registered with the Pan African Clinical Trial registry (PACTR). Registration no: PACTR202008784222254.

## Results

**Fig 1:**
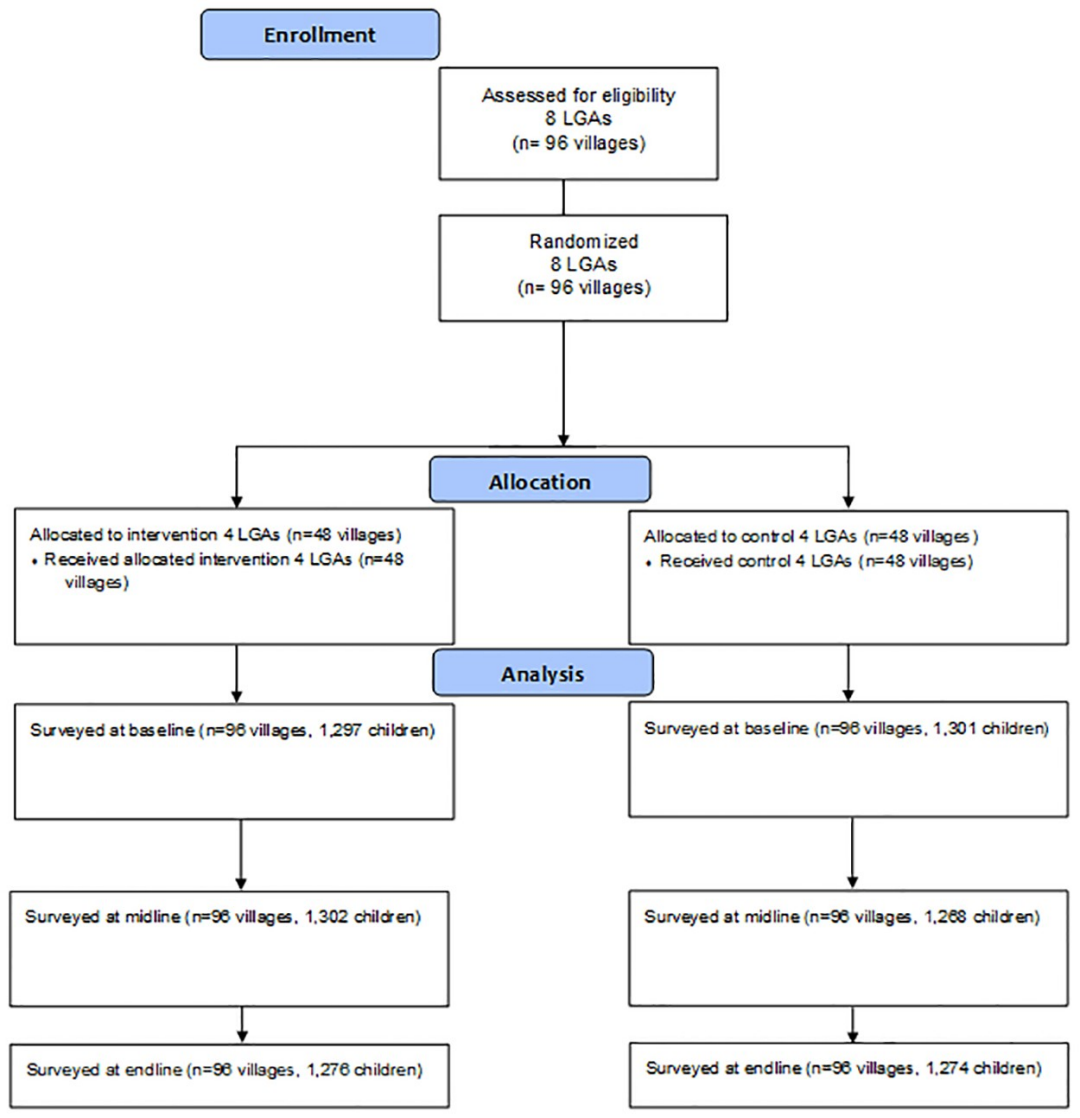
Study CONSORT flow diagram.

A total of 2598 children were recruited into the study at baseline, 2570 in the mid-line and 2550 in the final evaluation, slightly exceeding the target sample size of 2400 due to field logistics (Fig 1).

### Demographic characteristics of respondents

The characteristics of the respondents were similar in both the intervention and control arms (Table 1), except for whether they lived in hard-to-reach communities or not. The age distributions of the children were also similar (Table 2).

**Table 1:** Characteristics of the respondents.

	Control Baseline n = 1301	Control Mid-term n = 1268	Control Final n = 1274	Intervention Baseline n = 1297	Intervention Mid-term n = 1302	Intervention Final n = 1276
*age in years*						
13–19	107 (8%)	89 (7%)	89 (7%)	92 (7%)	107 (8%)	78 (6%)
20–29	702 (54%)	690 (54%)	727 (57%)	765 (59%)	782 (60%)	770 (60%)
30–39	434 (33%)	444 (35%)	413 (32%)	372 (29%)	374 (29%)	396 (31%)
40–49	48 (3.7%)	37 (3%)	40 (3%)	51 (3.9%)	29 (2%)	32 (3%)
50–59	7 (0.5%)	5 (0.4%)	3 (0.2%)	12 (0.9%)	9 (0.6%)	0
60+	2 (0.2%)	2 (0.2%)	2 (0.2%)	5 (0.4%)	1 (0.07%)	0
age not known**	1 (0.1%)	1(0.1%)	0	0	0	0
*Level of education of caregivers*						
None	30 (2%)	30 (2%)	21 (2%)	14 (1%)	12 (0.9%)	8 (0.6%)
Primary	248 (19%)	223 (18%)	221 (17%)	168 (13%)	158 (12%)	107 (8%)
Secondary	818 (63%)	848 (67%)	857 (67%)	973 (75%)	932 (72%)	939 (74%)
Tertiary	205 (16%)	167 (13%)	175 (14%)	142 (11%)	200 (15%)	222 (17%)
*Religious affiliation*						
Orthodox	679 (52%)	707 (56%)	826 (65%)	726 (56%)	780 (60%)	757 (59%)
Pentecostal	590 (45%)	528 (42%)	410 (32%)	543 (42%)	496 (38%)	486 (38%)
White garment	18 (1%)	15 (1%)	14 (1%)	21 (2%)	20 (2%)	25 (2%)
Islam/Others/None	14 (1%)	18 (1%)	24 (2%)	7 (0.5%)	6 (0.4%)	8 (0.6%)
*Where help was sought last for child’s ill health*						
Health facility	674 (58%)	649 (53%)	404 (38%)	568 (47%)	624 (53%)	554 (46%)
Medicine shop	318 (27%)	372 (31%)	500 (47%)	580 (48%)	430 (36%)	557 (46%)
Treated at home: drugs	146 (13%)	146 (12%)	120 (11%)	31 (3%)	85 (7%)	63 (5%)
Treated by a friend: drugs	19 (2%)	15 (1%)	10 (0.9%)	5 (0.4%)	15 (1%)	12 (1%)
Other***	10 (1%)	35 (3%)	26 (2%)	14 (1%)	28 (2%)	19 (2%)
*Distance to health facility*						
15min walk or less	452 (35%)	559 (44%)	618 (49%)	491 (38%)	513 (39%)	567 (44%)
15-<30min	458 (35%)	346 (27%)	364 (29%)	506 (39%)	580 (45%)	431 (34%)
30-<45m	136 (10%)	129 (10%)	115 (9%)	188 (14%)	118 (9%)	194 (15%)
45m-1h	107 (8%)	137 (11%)	104 (8%)	74 (6%)	53 (4%)	58 (5%)
>1h	148 (11%)	97 (8%)	73 (6%)	38 (3%)	38 (3%)	26 (3%)
*Hard to reach*						
Yes	131 (10%)	87 (7%)	83 (7%)	539 (42%)	602 (46%)	572 (45%)
No	1170 (90%)	1181 (93%)	1191 (93%)	758 (58%)	700 (54%)	704 (55%)

NOTE:

** percentages of known values (excluding missing values).

*** Included only ‘other’ that had been ill—some of the answers to this question suggested that the child had not been ill.

**Table 2:** Characteristics of the children by survey.

	Control baseline N = 1301	Control Mid-term N = 1268	Control final N = 1274	Intervention baseline N = 1297	Intervention mid-term N = 1302	Intervention Final N = 1276
*Age of child in months*						
0–5	460 (35%)	406 (32%)	410 (32%)	468 (36%)	507 (39%)	429 (34%)
6–11	321 (25%)	338 (27%)	377 (30%)	313 (24%)	382 (29%)	405 (32%)
12–17	302 (23%)	311 (25%)	294 (23%)	296 (23%)	238 (18%)	244 (19%)
18–23	218 (17%)	213 (17%)	193 (15%)	220 (17%)	175 (13%)	198 (16%)
*Sex of child*						
Female	651 (50%)	618 (49%)	629 (49%)	648 (50%)	649 (50%)	654 (51%)
Male	650 (50%)	650 (51%)	645 (51%)	649 (50%)	653 (50%)	622 (49%)
*Birth order*						
First	401 (31%)	378 (30%)	386 (30%)	416 (32%)	384 (30%)	369 (29%)
Second	356 (27%)	335 (26%)	329 (26%)	333 (26%)	340 (26%)	347 (27%)
Third	257 (20%)	248 (20%)	252 (20%)	245 (19%)	241 (19%)	285 (22%)
Fourth	142 (11%)	153 (12%)	139 (11%)	129 (10%)	154 (12%)	140 (11%)
Fifth	73 (6%)	76 (6%)	80 (6%)	97 (7%)	98 (8%)	77 (6%)
Sixth	39 (3%)	31 (2%)	46 (4%)	54 (4%)	54 (4%)	43 (3%)
other birth order	33 (3%)	47 (4%)	42 (3%)	23 (2%)	31 (2%)	15 (1%)

The proportion of children who were up-to-date with vaccinations increased slightly in both the intervention and control groups (Table 3). There was no evidence of an effect of the intervention on being up-to-date (OR at the endline survey 0.95 (95% CI 0.75–1.21); p = 0.69) (Table 4). The proportion of children who had had no vaccinations decreased over time from 7% to 0.4% in the intervention arm but there was no apparent decrease in the control arm (Table 3). The intervention increased the odds of the child having at least one vaccine at the final survey (OR: 12.13(95% CI 6.03–24.41); p <0.001) (Table 4).

**Table 3:** Vaccination status of children in the cross-sectional surveys.

	Control baseline	Control mid-term	Control Final	Intervention baseline	Intervention mid-term	Intervention final
	N = 1301	N = 1268	N = 1274	N = 1297	N = 1302	N = 1276
Not vaccinated	125 (10%)	104 (8%)	128 (10%)	87 (7%)	25 (2%)	5 (0.4%)
Partial	551 (42%)	452 (36%)	449 (35%)	619 (48%)	574 (44%)	610 (48%)
Up-to-date	625 (48%)	712 (56%)	697 (55%)	591 (46%)	703 (54%)	661 (52%)

**Table 4:** Estimated Impact of the intervention.

	Mid-survey vs baseline		Endline survey vs baseline	
	OR (95% CI)	p-value	OR (95% CI)	p-value
Up-to-date vs partial & not vaccinated	0.96 (0.76–1.22)	0.74	0.95 (0.75–1.21)	0.69
Up-to-date & partial vs not vaccinated	2.21 (1.37–3.57)	0.001	12.13(6.03–24.41)	<0.001

NOTE: The analysis was carried out using logistic regression with random effects for LGA, Ward and village to take account of clustering in the sample. The effect of the intervention was estimated as the difference in the change from baseline to the survey under consideration in the intervention arm compared to the change from baseline to the survey in the control arm. Each vaccine had 1.5% to 2% respondents who said they did not know if the child had received the vaccine: for the purposes of calculating vaccine status, we counted these as not having had the vaccine.

### Outcomes and estimation

We used interaction tests to assess whether there was a difference in the effect of the intervention by age group, stratification zone, distance to the health facility and “hard-to-reach” and found no consistent evidence of any interactions (p>0.05 for both up-to-date and partial compared to not vaccinated). During the baseline survey, the vaccination cards of around 70% of children were seen by the interviewer. While this remained at 70% in the control arm, it increased in the intervention arm to roughly 90% in the mid-line and final surveys.

Less than 50 percent of the children received Pentavalent 1 on time in both arms of the study at baseline (Table 5). There was a 3% decrease in the control arm, the intervention arm increased by 14% at end line evaluation: the estimated effect of the intervention was statistically significant (OR 1.96; 95% CI 1.53–2.53; p <0.001). Similarly, the proportion with timely uptake of Pentavalent 3 decreased by 4%in the control arm, and in the intervention arm there was an increase of 5% (OR 1.55; 95% CI: 1.14–2.12; p = 0.005). Measles’ timely vaccination remained the same in the control arm at the end of the intervention but there was almost a three-fold increase in the odds of receiving measles vaccine on time associated with the intervention (OR 2.81; 95% CI: 1.93–4.1; p<0.001) (Table 5).

**Table 5:** Proportion that had timely pentavalent and measles vaccination.

	Control baseline	Control mid-term	Control final	Intervention baseline	Intervention mid-term	Intervention final	mid-term OR95% CI p-value	final OR95% CI p-value
Pentavalent 1	531 (46%)	529 (46%)	496 (43%)	511 (46%)	632 (57%)	694 (60%)	1.63 (1.26–2.09) <0.001	1.96 (1.53–2.53) <0.001
Pentavalent 2	375 (36%)	377 (35%)	341 (32%)	340 (33%)	450 (44%)	447 (41%)	1.69 (1.29–2.22) <0.001	1.63 (1.25–2.14) <0.001
Pentavalent 3	273 (28%)	272 (27%)	243 (24%)	226 (24%)	311 (33%)	292 (29%)	1.72 (1.26–2.35) <0.001	1.55 (1.14–2.12) 0.005
Measles	155 (24%)	158 (25%)	154 (24%)	124 (19%)	211 (37%)	240 (41%)	2.53 (1.73–3.68) <0.001	2.81 (1.93–4.10) <0.001
Pentavalent 3 on time of those who had pentavalent 1 on time	254 (59%)	258 (57%)	239 (57%)	214 (50%)	295(55%)	281 (48%)	1.15 (0.77–1.74) 0.44	1.20 (0.80–1.81) 0.37

NOTE:

*Timeliness defined as within 2 weeks before or after target age.

At baseline, 1300 (85%) of the children who had had Pentavalent 1 and were aged at least 16 weeks went on to have Pentavalent 3. We estimated the effect of the intervention on children having Pentavalent 3 given that they had had Pentavalent 1. There was a small but significant effect on dropout: there was an increase in the proportion with Pentavalent 3 by the endline survey (midterm (OR = 1.21 (95% CI 0.80–1.84) p = 0.36) and endline (1.66 (95% CI 1.08–2.55) p = 0.02).

The intervention was significantly associated with an increase in the proportion of children with mother who had received two or more doses of tetanus toxoid, and attended ANC, and a decrease in the proportion of children reported to have had measles at the final but not at the midterm survey (Table 6).

**Table 6:** Health-care utilisation by the mother, and child illness.

	Control Baseline n = 1301	Control Mid-term n = 1268	Control Final n = 1274	Intervention Baseline n = 1297	Intervention Mid-term n = 1302	Intervention Final n = 1276	p-value
*Tetanus vaccination status of mother*							
None	195 (15%)	189 (15%)	168 (13%)	131 (10%)	101 (8%)	60 (5%)	
One	117 (9%)	123 (10%)	136 (11%)	144 (11%)	148 (11%)	146 (11%)	
Two	717 (55%)	692 (55%)	770 (60%)	753 (58%)	771 (59%)	813 (64%)	0.28^a^^c^
Three	237 (18%)	229 (18%)	182 (14%)	256 (20%)	258 (20%)	240 (19%)	0.02^b^^c^
More than three	35 (3%)	35 (3%)	18 (1%)	13 (1%)	24 (2%)	17 (1%)	
*Attendance at ANC*							
Yes	1114 (86%)	1129 (89%)	1143 (90%)	1148 (89%)	1206 (93%)	1240 (97%)	0.95^a^
No	183 (14%)	137 (11%)	131 (10%)	134 (10%)	95 (7%)	36 (3%)	<0.001^b^
Don’t know	4 (0.3%)	2 (0.1%)	0	15 (1%)	1 (0.08%)	0	
*Has the child ever been ill with measles*?							
Yes	1229 (95%)	1238 (98%)	1227 (97%)	1178 (91%)	1259 (97%)	1252 (99%)	0.37^a^
No	71 (5%)	28 (2%)	43 (3%)	115 (9%)	35 (3%)	18 (1%)	<0.001^b^
Not known	1	2	4	4	8	6	

NOTE: Percentages are of known values (excluding missing values).

^a^Effect of intervention on change between baseline and mid-term surveys.

^b^Effect of intervention on change between baseline and final survey.

^c^Comparing 0–1 vs 2 or more tetanus doses. In all analyses, missing values were excluded.

The number of reported suspected measles cases was extracted from the DHIS. The numbers were small, and the trends similar in the two arms. There was no report on deaths from the vaccine preventable disease. The trends in facility attendance for general outpatient, antenatal care, and delivery were similar in the two arms.

## Discussion

Several studies have assessed the impact of various interventions on full vaccination coverage among children less than two years of age. These included monetary incentives [15, 16], and the provision of monthly reliable vaccination service [17]. In a systematic review that included these studies, an intervention using non-monetary incentives improved full vaccination (OR 6.6; 95% CI3.93–11.28); ensuring the availability of vaccination service through outreach also had a positive impact on full vaccination (RR 3.09, 95% CI 1.69–5.67). Monetary incentives, on the other hand, had little or no effect (RR 1.03, 95% CI 0.83–1.28) in improving full coverage of vaccination in children [18]. Our study showed no evidence of an effect of the multi-faceted intervention using traditional and religious leaders on the proportion of children up-to-date with vaccination. The observed difference in the impact of the interventions may be due to differences in the interventions themselves. While the monetary incentive study examined conditional cash transfers targeting poverty reduction, our study targeted improving the knowledge of the community gate keepers (leaders) to influence their communities. These two studies could be said to be interventions that support “pulling” (demand for service) from the recipients. The non-monetary incentive on the other hand (a reward to caregivers for attending the health facility) and the outreach directly targeted vaccination services and supported “pushing” (supply of the services) to the recipients. It may be that interventions that directly target vaccination services are more likely to improve full vaccination coverage. Outreach, in particular, has been reported to improve interactions between the caregiver and the health worker, thereby enhancing vaccination uptake [19].

On the other hand, delayed vaccination in our study setting, which could be attributed to the weak health system, could have contributed to the failure of achieving full coverage of vaccination. Immunisation services were provided on a monthly basis in most of the facilities studied. It could be inferred that if the health system is strengthened to provide more frequent services, the possibility of achieving full vaccination coverage with the intervention will be high, particularly as the non-vaccinated children were reached. While the sustainability of the monetary incentive is questionable, our intervention has a good chance of being sustainable as it is embedded into an existing structure.

The intervention was found to be effective in increasing the proportion of children receiving at least one vaccination; and improved timeliness of vaccination for all vaccines. A review of grey literature has shown parental attitudes and knowledge play a major role in the lack of vaccination of children [20]. The TRL intervention provides an opportunity to impact on parental knowledge and attitude through the community gatekeepers. A meta-analysis of the impact of other community-based health education interventions on increase in DTP3 uptake was reported [18].

The reduction in the dropout in Pentavalent 3 vaccination may be partly due to the improved timeliness of vaccination. Reasons for DTP3 dropout have been attributed to demand side factors rather than supply side factors in rural India [21]. To reduce DTP3 dropout rate, interventions to drive the demand for vaccination should be considered.

Data from the survey showed that the intervention may have affected the level of utilization of the health facilities by mothers. Mothers in the intervention arm were more likely to attend ANC and to have received at least two doses of tetanus toxoid during pregnancy.

Cases of measles declined during the study in both arms, shown in both the proportion of children with reported illness and in the DHIS trends over time. This observation could be attributed to the Measles campaign that was carried out across all the LGAs in the State in March 2017; Measles vaccine was administered to children aged 9 months to 5 years during the campaign. The intervention was introduced in May 2017 and there was evidence of an impact of the intervention: it appeared to have sustained the gains of the Measles campaign in the intervention arm of the study. The follow-up time was short to confirm this.

The training of the health workers may have contributed to the increase in the number of vaccination cards seen in the intervention arm at midline and endline surveys and the improved vaccine uptake. It may also have impacted on the outcomes, but it is difficult to ascertain the direction of the impact since, rather than improve the completion of vaccination it reduced the number of unvaccinated children. The non-functionality of most of the ward development committees in the intervention arm of the study before the intervention makes sustainability of the intervention doubtful in such setting as they may lapse back into inactivity. It is also possible that the impact of the intervention could have been greater among Wards with self-motivated development committees.

The TRLs can be seen as the untapped resources in the community that the implementers and practitioners can take advantage of to boost and sustain vaccination coverage. It is feasible where the traditional and religious leaders are key influencers in their communities. It is likely to be more impactful where the traditional and religious leadership are embedded in one system. Though the intervention was not effective in improving the proportion of children up-to-date on vaccination, several other outcomes were impacted on especially reduction in the proportion of the unvaccinated children. An intervention that reduces the proportion of the unvaccinated children is of importance particularly in populous nations who contribute more of the unvaccinated children. It is, therefore, important that health workers involved in vaccination engage the TRLs actively for routine vaccination in such settings. For a child to be up-to-date with vaccination, the health system needs to be strengthened to ensure regular access to vaccination services. The synergy between different strategies to achieve the Global Vaccine coverage goal cannot be over emphasized.

### Study limitations

More vaccination cards were seen in the intervention arm at midterm and end line evaluation. It is possible that there was differential bias though the direction is difficult to ascertain. Since the proportion of vaccination cards tended to increase with the intervention but remained the same in the control arm, the estimated effect of the intervention may potentially have a bias.

## Supporting information

S1 Checklist (DOCX)

S1 Fig (TIF)
