# Supplementary figures and images for: Effects of engaging communities in decision-making and action through traditional and religious leaders on vaccination coverage in Cross River State, Nigeria: A cluster-randomised control trial
Source: PLoS One. 2021 Apr 16;16(4):e0248236. doi: 10.1371/journal.pone.0248236 (PMC8051768; doi:10.1371/journal.pone.0248236)

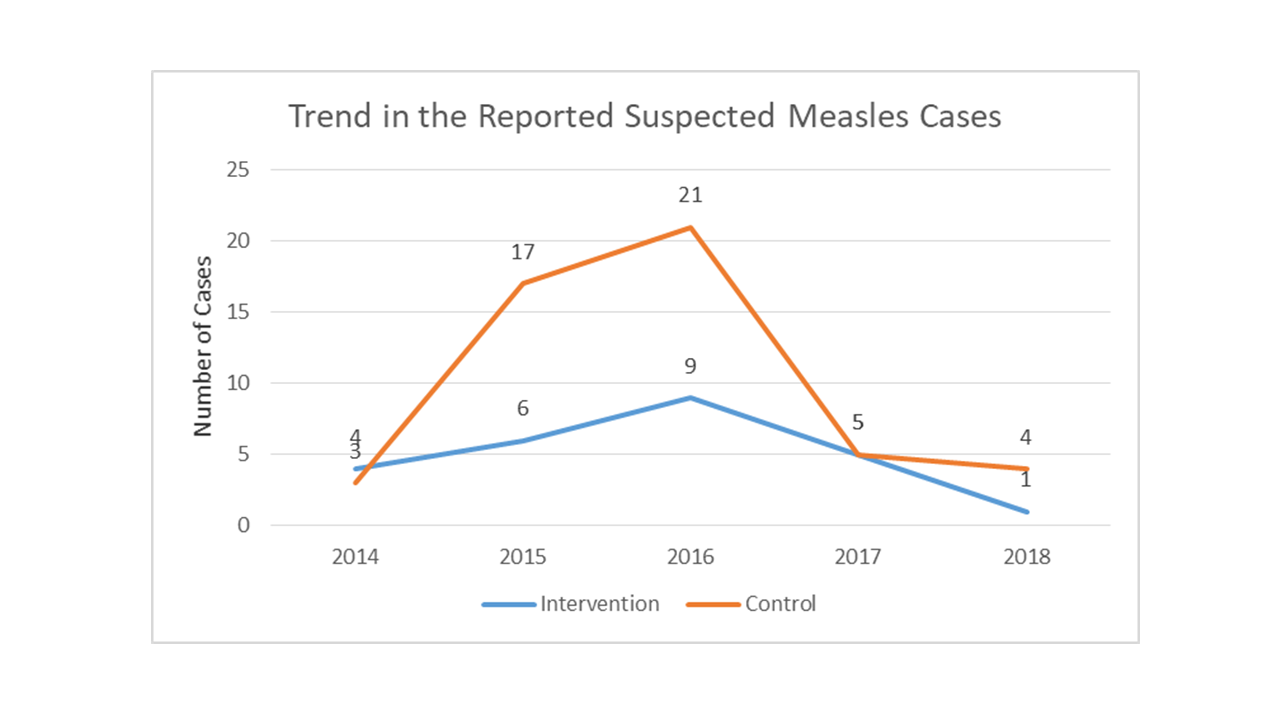

Supplement: S1 Fig — (TIF) [file pone.0248236.s002.tif]
